# Reemergence of Dengue in Southern Texas, 2013

**DOI:** 10.3201/eid2206.152000

**Published:** 2016-06

**Authors:** Dana L. Thomas, Gilberto A. Santiago, Roman Abeyta, Steven Hinojosa, Brenda Torres-Velasquez, Jessica K. Adam, Nicole Evert, Elba Caraballo, Elizabeth Hunsperger, Jorge L. Muñoz-Jordán, Brian Smith, Alison Banicki, Kay M. Tomashek, Linda Gaul, Tyler M. Sharp

**Affiliations:** Centers for Disease Control and Prevention, San Juan, Puerto Rico (D.L. Thomas, G.A. Santiago, B. Torres-Velasquez, E. Caraballo, E. Hunsperger, J.L. Muñoz-Jordán, K.M. Tomashek, T.M. Sharp);; Centers for Disease Control and Prevention, Atlanta, Georgia, USA (D.L. Thomas, J.K. Adam);; Cameron County Health Department, Harlingen, Texas, USA (R. Abeyta);; Texas Department of State Health Services, Harlingen (R. Abeyta, B. Smith);; Hidalgo County Health and Human Services, Edinburg, Texas, USA (S. Hinojosa);; Division of Global Migration and Quarantine, San Diego, California, USA (J.K. Adam);; Texas Department of State Health Services, Austin, Texas, USA (N. Evert, A. Banicki, L. Gaul)

**Keywords:** Dengue, United States, Texas, outbreak, viruses, vector-borne infections, reemergence, dengue virus

## Abstract

During a dengue epidemic in northern Mexico, enhanced surveillance identified 53 laboratory-positive cases in southern Texas; 26 (49%) patients acquired the infection locally, and 29 (55%) were hospitalized. Of 83 patient specimens that were initially IgM negative according to ELISA performed at a commercial laboratory, 14 (17%) were dengue virus positive by real-time reverse transcription PCR performed at the Centers for Disease Control and Prevention. Dengue virus types 1 and 3 were identified, and molecular phylogenetic analysis demonstrated close identity with viruses that had recently circulated in Mexico and Central America. Of 51 household members of 22 dengue case-patients who participated in household investigations, 6 (12%) had been recently infected with a dengue virus and reported no recent travel, suggesting intrahousehold transmission. One household member reported having a recent illness consistent with dengue. This outbreak reinforces emergence of dengue in southern Texas, particularly when incidence is high in northern Mexico.

## Abstract 2

Of 53 cases detected, about half were acquired locally.

Dengue is an acute febrile illness that is common throughout the tropics and subtropics (*1*) and is a leading cause of febrile illness in travelers returning to the United States from these regions (*2*). From the late 1700s until the 1940s, dengue outbreaks occurred regularly in the southern United States (*3*) but did not occur after a campaign that rid much of the continental United States of *Aedes aegypti* mosquitoes, which transmit the 4 dengue virus types (DENV-1–4) (*4*). After the campaign ended, however, *Ae. aegypti* mosquito populations soon resurged (*5*), and dengue reemerged in southern Texas in 1980 (*6*). The 1980 dengue outbreak and subsequent outbreaks in 1999 (*7*) and 2005 (*8*) were associated with epidemics in northern Mexico. During the 1999 outbreak, despite a higher prevalence of mosquito-infested water containers in Texas, DENV infection was less frequent among residents of Texas than northern Mexico, partly because of the more prevalent use of air-conditioning, window screens, and other factors that limit human–mosquito contact in Texas (*7*). In line with these observations, infection during the 2005 outbreak was also associated with lower socioeconomic status (*8*).

During 2013, a dengue epidemic occurred in northern Mexico; >5,500 dengue cases were reported from the state of Tamaulipas (*9*), which shares a border with Texas. The first laboratory-positive dengue case in Texas was reported to the Texas Department of State Health Services in July, during the peak of the Tamaulipas epidemic. To identify and describe all suspected dengue cases in southern Texas (Cameron, Hidalgo, Starr, and Willacy Counties), enhanced dengue surveillance was conducted; this surveillance included case finding and additional diagnostic testing of specimens from suspected dengue patients. To describe the molecular epidemiology of the DENVs responsible for the outbreak, we performed molecular phylogenetic analysis. To identify persons with subclinical (either asymptomatic or symptomatic but not medically assessed) DENV infection and to describe demographic or behavioral factors associated with intrahousehold DENV transmission, we also conducted household investigations of patients with laboratory-positive dengue. This investigation proposal underwent review at the Centers for Disease Control and Prevention (CDC; Atlanta, GA, USA) and was determined to be public health practice and not research; as such, institutional review board approval was not required.

## Methods

We identified dengue cases by compiling Texas Department of State Health Services surveillance case reports, retrieving positive and negative dengue diagnostic test results from 2 commercial laboratories, and conducting medical record reviews for patients for whom dengue diagnostic testing had been ordered. Specimens submitted to commercial laboratories were tested by IgM ELISA according to internal protocols. Available specimens from commercial laboratories were forwarded to CDC for testing by real-time reverse transcription PCR (rRT-PCR) (*9*).

For all specimens that were positive by rRT-PCR, we attempted amplification of the envelope glycoprotein gene (1,485 bp), followed by Sanger bidirectional sequencing; phylogenetic relationships were inferred by using previously described methods (*10*). We sequenced 5 DENV-1 isolates and 1 DENV-3 isolate. Bayesian maximum clade credibility trees were inferred to estimate genotype and phylogenetic origins.

Dengue case-patients and their household members were offered participation in household investigations. Interviews were conducted during November 2013–January 2014 and within 90 days of reported illness onset for the index case-patients. Household members completed a questionnaire that collected information on demographic, behavioral, and household characteristics; they also provided a blood specimen for dengue diagnostic testing by rRT-PCR and anti-DENV IgM antibody-capture (MAC) ELISA (InBios International, Inc., Seattle, WA, USA) performed at CDC.

We defined a suspected dengue case-patient as a patient with an acute febrile illness who sought medical care for which a clinician ordered dengue diagnostic testing. We defined a laboratory-positive case-patient as a patient with suspected dengue and a positive test result by rRT-PCR or by IgM or MAC ELISA. Because the day of specimen collection after illness onset was unknown for most patients tested at private laboratories, we defined a laboratory-negative case-patient as a patient with suspected dengue and negative IgM ELISA and rRT-PCR results for the same specimen or 2 negative IgM ELISA results for different specimens. We defined a laboratory-indeterminate case-patient as a patient with suspected dengue and a single negative IgM ELISA result. Intrahousehold DENV transmission (i.e., presumed mosquito-transmitted DENV infection within or near the home of a laboratory-positive case-patient) was defined by detection of DENV infection by rRT-PCR or MAC ELISA in a household member who had not traveled to Mexico in the past 90 days. Dengue and dengue hemorrhagic fever were defined according to the 1997 World Health Organization guidelines (*11*). 

## Results

**Figure 1:**
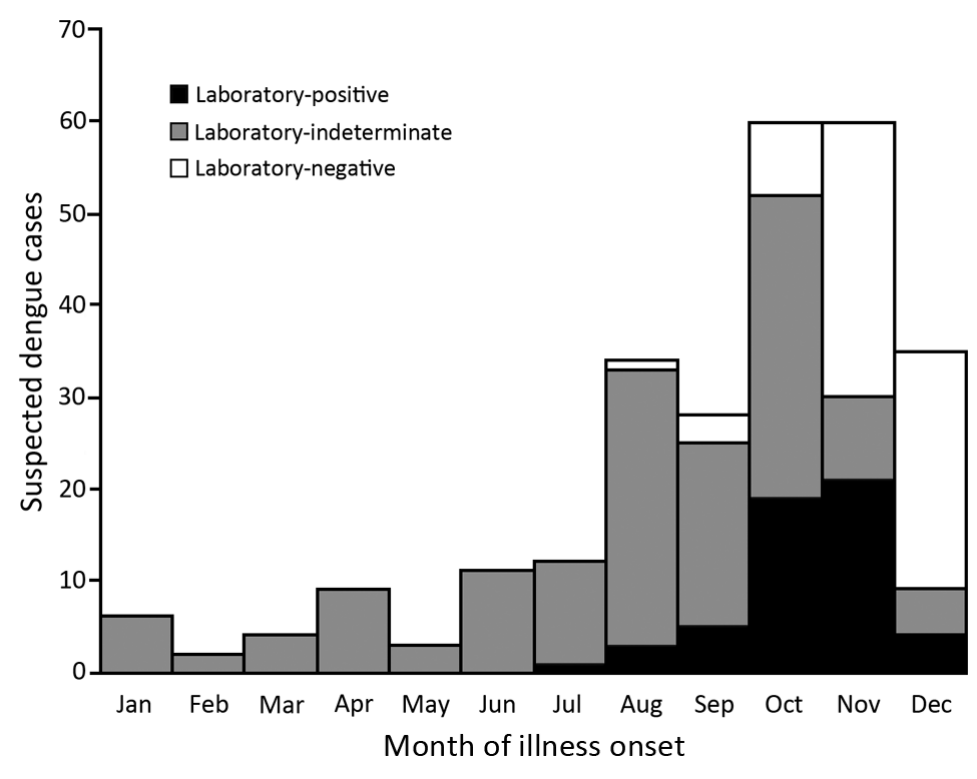
Suspected dengue cases identified by the Texas Department of State Health Services, 2013. A total of 264 suspected dengue cases were reported along with IgM ELISA diagnostic test results obtained from commercial diagnostic laboratories. A subset of 112 available specimens was forwarded for confirmatory diagnostic testing by real-time reverse transcription PCR and anti–dengue virus IgM ELISA. Black, positive result (n = 53); gray, laboratory-indeterminate result (n = 127); white, laboratory-negative result (n = 84).

Of 264 suspected dengue cases identified in southern Texas during 2013, a total of 53 (20%) were laboratory-positive; 24 (45%) of the laboratory-positive cases had been reported to the Texas Department of State Health Services. Suspected cases were identified throughout the year; about half (47%) occurred in October and November, when one third of suspected cases were laboratory-positive (Figure 1). A total of 112 serum specimens were forwarded from commercial laboratories to CDC for additional diagnostic testing by rRT-PCR; most of these specimens came from patients whose illness began during October–December. Positive rRT-PCR results were obtained for 14 (17%) of 83 IgM-negative specimens and 8 (28%) of 29 IgM-positive specimens. Of the 22 specimens positive by rRT-PCR, DENV-1 was detected in 19 (86%) and DENV-3 in 3 (14%).

**Figure 2:**
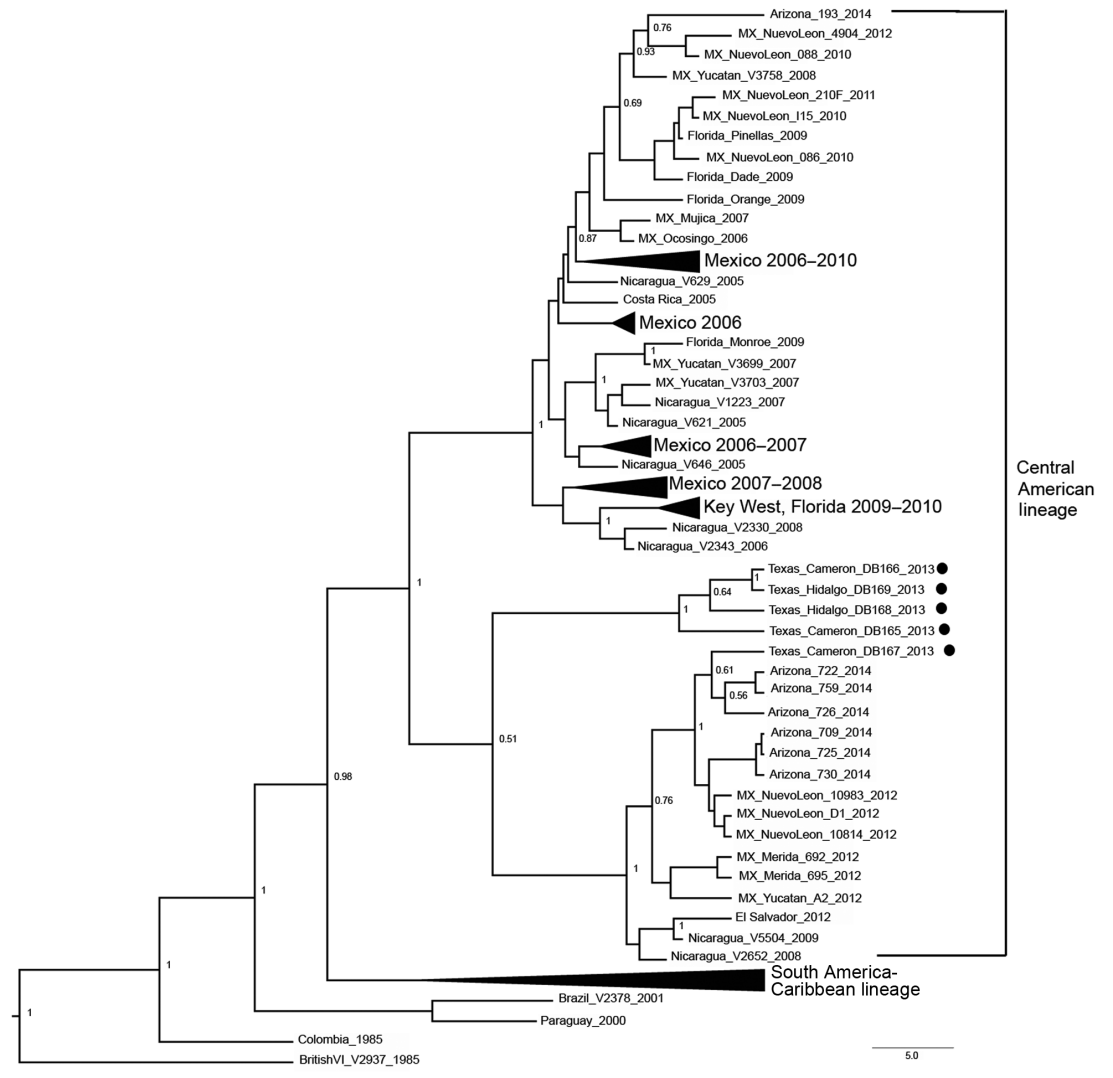
Phylogenetic tree of the 5 dengue virus type 1 isolates obtained from dengue case-patients in southern Texas, 2013. Maximum clade credibility tree inferred from 90 envelope glycoprotein gene sequences: 5 from Texas in 2013 (solid circles), and 85 from GenBank. BEAST version 1.8.2 (http://beast.bio.ed.ac.uk/) was used with strict molecular clock constant population size and 10 million Markov chain Monte Carlo iterations; effective sample size >200. Posterior probabilities >0.50 are shown in major nodes. All sequences shown belong to the American-African genotype. Other genotypes were not included to increase resolution of the Central American lineage. Black tapered lines indicate condensation of a monophyletic lineage with a single common ancestor. Scale bar indicates nucleotide substitutions per site. MX, Mexico.

All sequenced DENV-1 isolates belonged to the American-African genotype and diverged from a distinct lineage of Central American origin (Figure 2). The sequenced isolates clustered together in a subclade associated with contemporary sequences from viruses isolated in Nuevo Leon and the Yucatan Peninsula in Mexico, Nicaragua, El Salvador, and during an outbreak of travel-associated dengue in southern Arizona that occurred in late 2014 (*12*). Of the 5 Texas DENV-1 sequences, 4 diverged into a separate cluster within this subclade, and the fifth sequence was closely associated with a cluster from Nuevo Leon, Mexico, in 2012. The DENV-3 isolate belonged to the Indian subcontinent genotype, which commonly circulates in the Americas, and clustered with other contemporary sequences from viruses isolated in Nicaragua and El Salvador (data not shown).

Of the 53 laboratory-positive case-patients, most (70%) resided in Cameron County, 15 (28%) in Hidalgo County, and 1 (2%) in Willacy County. Median case-patient age was 28 years (range 1–85 years), and more than half (58%) of case-patients were female. Of 49 case-patients who reported their travel history, 26 (53%) did not report travel outside of Texas in the 2 weeks before illness onset and the remainder reported recent travel to Mexico. The case definition for dengue was met by 52 (98%) cases, and the definition for dengue hemorrhagic fever was met by none. More than half (29 [55%]) of the 53 laboratory-positive case-patients were hospitalized.

Participation in the household investigations was agreed to by 22 (42%) laboratory-positive dengue case-patients from 22 households and by 51 (54%) of their 95 household members, none of whom had been reported as a suspected dengue case-patient. Evidence of recent DENV infection was found by MAC ELISA for 7 (14%) of the 51 household members; all were older than those without recent infection (Table). Five (71%) of the 7 household members with recent DENV infection reported neither recent travel outside of Texas nor febrile illness, suggesting locally acquired but asymptomatic DENV infection. One (14%) of the 7 household members with recent DENV infection, a 79-year-old woman, reported having had fever, headache, body and eye pain, nausea and vomiting, and anorexia that began 2 weeks after her husband, the case-patient, became ill. She sought medical care for her illness in Mexico, where she was hospitalized for persistent vomiting and abdominal pain. After a 3-day hospitalization, she was discharged home in good condition.

**Table:** Characteristics of households and household members with evidence of intrahousehold transmission of DENV, southern Texas, 2013*

Characteristics	No evidence of intrahousehold transmission, n = 9†	Evidence of intrahousehold transmission, n = 6†
Households		
No. residents, mean (range)	2 (2–6)	4 (3–5)
Ratio of adults:children, mean (range)	0.8 (0.2–1)	2.8 (1–4)
Recent visitors from Mexico, no. (%)‡	4 (44)	1 (17)
Window screens, no. (%)	8 (89)	4 (67)
Air-conditioning, no. (%)	7 (78)	6 (100)
Mosquitoes recently seen in house, no. (%)	7 (78)	2 (33)
Household members	No evidence of recent DENV infection, n = 44	Evidence of recent DENV infection, n = 7
Age, y, median (range)	26 (1.4–71)	45 (3–79)
Portion of life lived in Mexico, mean % (range)	42 (0–96)	33 (0–71)
Recent travel to Mexico, no. (%)‡	25 (57)	1 (14)
Recently used mosquito repellant, no. (%)‡	27 (61)	4 (57)
Recent febrile illness, no. (%)‡	0 (0)	1 (14)

*DENV, dengue virus. †7 households were excluded from analysis because not all household members provided a serum specimen for diagnostic testing and therefore could not be confidently defined as not having evidence of intrahousehold transmission. ‡Within past 3 mo.

The 7 household members with evidence of recent DENV infection resided in 6 households that had more household members and more adults than those without evidence of intrahousehold DENV transmission (Table). Other household characteristics were similar between households with and without evidence of intrahousehold DENV transmission, including frequency of having a visitor from Mexico and having air-conditioning and window screens.

## Discussion

Consistent with previously reported outbreaks (*6*–*8*), the 2013 dengue outbreak in southern Texas occurred concurrently with an epidemic in Tamaulipas, Mexico. Enhanced surveillance enabled identification of 53 dengue cases in southern Texas, of which nearly one fifth initially had negative results from private laboratories and about half of the infections were locally acquired. This finding represents the largest number of locally acquired dengue cases in a single outbreak since dengue first reemerged in Texas in 1980. Molecular phylogenetic analysis of isolated DENVs determined that the viruses circulating in northern Mexico and southern Texas in 2013 were closely related to viruses that had recently circulated in Mexico and Central America. Investigations of households of dengue patients enabled identification of 7 additional persons who had recently been infected with DENV; 6 of these infections probably resulted from intrahousehold DENV transmission, and 1 was associated with an illness that was consistent with dengue.

Fewer than half of the dengue cases identified in this investigation were reported to the Texas Department of State Health Services, partly because nearly 1 in 5 case-patients received a false-negative serologic diagnostic test result from commercial laboratories. The high proportion of false-positives were probably the result of serologic testing being ordered for a specimen collected during the first 5 days of illness. Clinicians should be aware that anti-DENV IgM is typically not detectable until 3–5 days after illness onset (*10*), whereas DENV nucleic acid is typically detectable by rRT-PCR for the first 5 days of illness (*9*). Clinicians should therefore be encouraged to request both molecular and serologic diagnostic testing for dengue patients.

Of 5 DENV-1 isolates that were sequenced, 2 were from patients with locally acquired DENV infection and 3 were from patients who had traveled to Mexico. One DENV-1 isolate was determined to be closely related to a virus isolated from Nuevo Leon, Mexico, in 2012. The remaining DENV-1 isolates grouped together and were probably descendants of a common ancestor from northern Central America and Mexico. This grouping of isolates from Mexico and Texas demonstrates that multiple DENV-1 strains were co-circulating in northern Mexico and southern Texas in 2013. Moreover, the phylogenetic, geographic, and temporal data suggest that transmission of this lineage of DENV-1 is moving northward from Central America. More virus isolates will be required to further ascertain the relationships of DENV-1 and DENV-3 circulating along the United States/Mexico border region.

Consistent with previously reported data (*13*), half of dengue case-patients in southern Texas were hospitalized during the 2013 outbreak. This hospitalization rate (55%) is higher than rates in areas where dengue is endemic (10%–20%) (*14*). Potential explanations for this rate of patient hospitalization include detection bias based on severity of dengue disease, overhospitalization of dengue patients, and appropriate hospitalization of patients who were older than those in dengue-endemic areas and who had concurrent conditions or warning signs for severe dengue.

A strength of this investigation included identification of cases by further testing specimens by rRT-PCR after initial testing by IgM ELISA. However, a limitation of this investigation was that not all specimens initially tested at commercial laboratories were available for retesting by rRT-PCR. Thus, the true number of cases that occurred in southern Texas in 2013 was probably underestimated. Also, because most DENV infections in dengue-endemic areas occur in and around the home of infected persons (*15*), the finding of other household members with evidence of recent DENV infection in one quarter of case-patients’ households was not unexpected. Although only 1 person with recent DENV infection had an illness consistent with dengue, such infections are still relevant to identify, because asymptomatically infected persons have recently been shown to play a role in DENV dissemination (*15*–*17*). Nonetheless, these and other observations of the frequency and characteristics of infected persons cannot be applied to the population of southern Texas because they were derived from a convenience sample of households where known dengue case-patients resided. Last, because we were unable to definitively determine where infection of household members occurred, some households defined as having evidence of intrahousehold transmission may have been misclassified.

In 2013, >27 million travelers crossed the Mexico border into southern Texas (*18*), where *Ae. aegypti* mosquito populations are established (*19*). Therefore, future dengue epidemics in northern Mexico are likely to result in local DENV transmission in southern Texas. Residents of southern Texas should therefore empty, cover, or dispose of mosquito breeding sites (e.g., discarded tires, rain barrels, buckets) and use mosquito repellent to avoid mosquito bites. Clinicians should order both molecular and serologic diagnostic testing for suspected dengue patients, and positive results should be reported to public health authorities. Additional information on recommended diagnostic algorithms and dengue patient clinical management is available from CDC (http://www.cdc.gov/dengue/training/cme.html).
